# Multiple Sequence Alignments as Tools for Protein Structure and Function Prediction

**DOI:** 10.1002/cfg.313

**Published:** 2003-07

**Authors:** Alfonso Valencia

**Affiliations:** Protein Design Group National Centre for Biotechnology CNB-CSIC Madrid Spain

## Abstract

Multiple sequence alignments have much to offer to the understanding of protein structure, evolution and function. We are developing approaches to use this information in predicting protein-binding specificity, intra-protein and protein-protein interactions, and in reconstructing protein interaction networks.


**Comparative and Functional Genomics**
*Comp Funct Genom* 2003; **4**: 424–427.
Published online in Wiley InterScience (www.interscience.wiley.com). **DOI:** 10.1002/cfg.313

**Conference Review**

### Multiple sequence alignments as tools for protein structure and function prediction

Alfonso Valencia*
*Protein Design Group, National Centre for Biotechnology, CNB-CSIC, Madrid, Spain*

**Correspondence to:*
*Alfonso Valencia, Protein Design Group, National Centre for Biotechnology, CNB-CSIC, Madrid, Spain.*
*E-mail: valencia@cnb.uam.es*

Received: 22 May 2003
Revised: 6 June 2003
Accepted: 6 June 2003

### Abstract

**Multiple sequence alignments have much to offer to the understanding of protein structure, evolution and function. We are developing approaches to use this information in predicting protein-binding specificity, intra-protein and protein-protein interactions, and in reconstructing protein interaction networks. Copyright © 2003 John Wiley & Sons, Ltd.**

**Keywords: multiple sequence alignments; protein structure; prediction; protein interactions; correlated mutations; interaction networks**

Multiple sequence alignments are a precious repository of the successful evolutionary strategies explored by proteins [24]. We are interested in the development of computational methods able to systematically recover part of this information. A first line of research addressed the detection of positions with patterns of variation characteristic of the internal structure of the corresponding protein families. These 'tree determinant residues' are distributed in close proximity to regions dedicated to specific molecular recognition (e.g. binding sites, protein interaction regions) [5]. Indeed, by manipulating these residues it is possible to modulate protein-binding specificity [1] (Figure 1). The possibility of using predicted specificity sites for the prediction of the corresponding molecular functions still remains unexplored. We have initiated a complementary route for the exploration of potential binding regions by training neural networks with proteins with known interaction sites and the corresponding multiple sequence alignments [9]. In the future, we would like to include in this neural network framework the information concerning 'tree-determinant' residues to improve the quality of the predictions of protein binding/specific sites with sequence, or with combinations of sequence and structural information.

A second line of research studies the small variations in multiple sequence alignments that may be presented by amino acids that act in association to maintain protein stability against random mutational drift. These correlated positions present a weak correlation with physical proximity in protein structures [11,15] and can be combined with other signals to predict three-dimensional contacts [7,8]. Interestingly, they seem to be more effective in the detection of protein–protein interactions than in the prediction of intra-protein contacts [17] and, as such, can be used to build models of protein complexes [2] (Figure 2).

Sparked by the growing interest in deciphering protein interaction networks (Table 1), we extended both approaches to the prediction of protein interaction partners. The 'mirror-tree' method [18] and the '*in-silico*-2-hybrid' method [19,23] are based on the concepts of tree-determinants and correlated mutations, respectively. Recently, we have systematically evaluated the reliability of the protein interaction network prediction methods, using our previous developments for directly assessing the presence of the interactions in published papers. The predictions compared include those generated by each one of these two methods for the *E. coli* genome, those by other previous published methods based on information about genome

Copyright © 2003 John Wiley & Sons, Ltd.

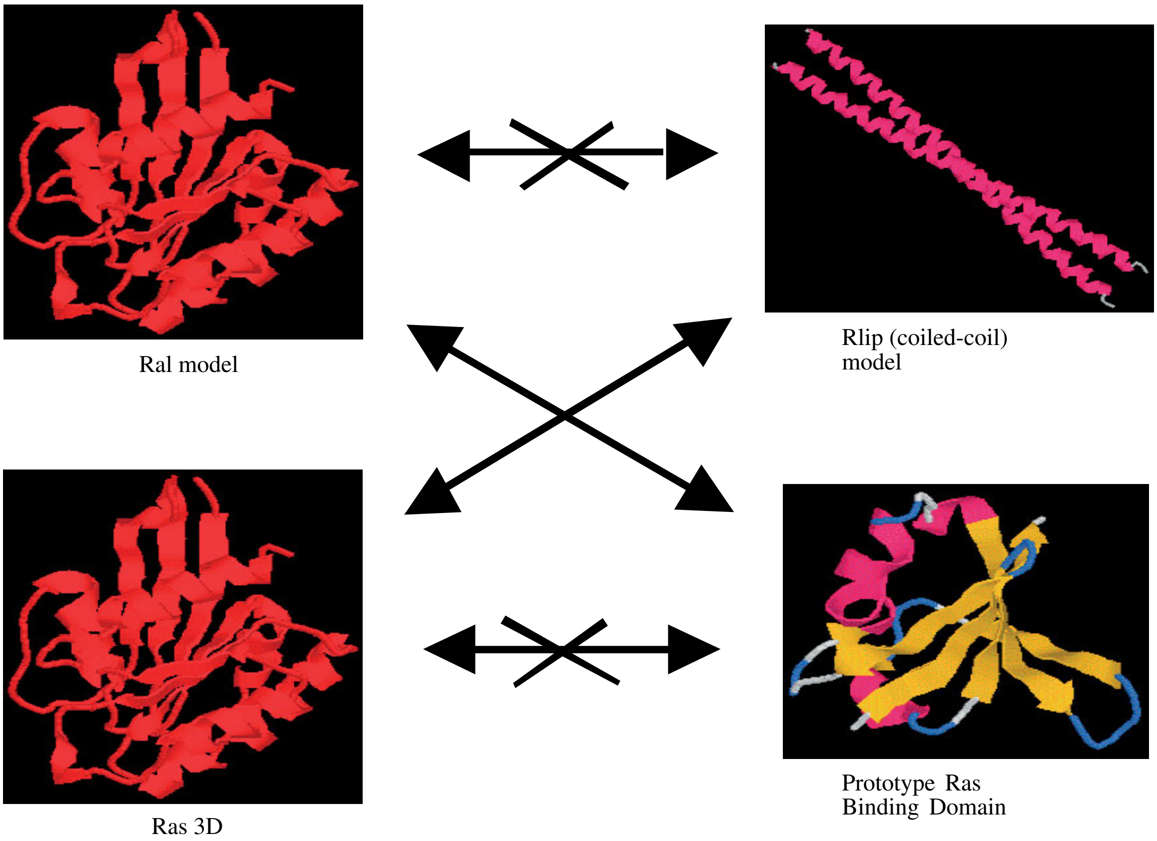

**Figure 1.** Swap of function (binding partner) between related proteins. Ras and Ral are very similar proteins of the larger ras super-family that bind to very different effectors: Rlip in the case of Ral, and Ras Binding Domain containing proteins for the ras proteins. The prediction of two residues key for the functional differentiation of the sequences was generated with the SequenceSpace software, based on the tendency of these two positions to contain information specific for each one of the two families [1]. The experimental exchange between the corresponding amino acids in these two positions produced a complete swap of the corresponding substrate-binding specificities. In this case the Ras double mutant bound Rlip and not the ras binding domain containing proteins and the Ral double mutant bound Ras Binding domain proteins and not Rlip [1]

**Table 1.** Current genome and sequence-based methods for the prediction of protein–protein interactions

| Method | References | Description |
|---|---|---|
| Gene neighbours | 3,16 | Uses the proximity of genes in bacterial genomes as criteria for the prediction of functional relations |
| Gene fusion | 6,13,14 | Explores the presence of fused genes producing a single peptide chain in some genomes for the prediction of interactions |
| Patterns of gene presence | 10,20 | Genes with a similar distribution in complete genomes are predicted to have related functions |
| Domain architecture | 12,22 | The domain composition of complex proteins is exploited for the prediction of associations between them |
| Mirror trees | 18 | The similarity of the gene trees of different protein families is quantified and used for the prediction of interactions |
| *In silico*-2-hybrid | 19 | The presence of 'correlated positions' between pairs of positions in pairs of multiple sequence alignments is used as indicative of their potential molecular interaction |

Copyright © 2003 John Wiley & Sons, Ltd. 

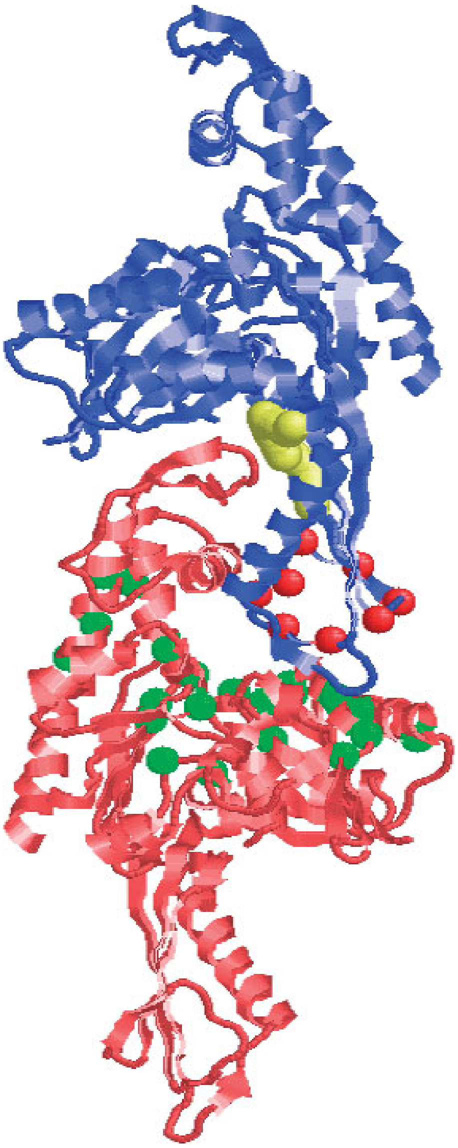

**Figure 2.** Proposed model of polymerization for FtsA. The residues participating in correlations are coloured red and green. The model was selected as the docking model that best fits the distance constraints imposed by the correlated residues. The model is compatible with the position of a peptide able to interrupt dimer formation, which corresponds to an interface region highlighted in yellow in the picture. Reproduced from [2] by permission of John Wiley and Sons Ltd organization [3,6,20], and a set of results based on an experimental two-hybrid approach [21]. Our results show [4] that all the computational methods have a similar coverage and accuracy, which are also similar to those obtained with the experimental approach. This analysis opens new possibilities for a combination of these methods for an effective reconstruction of protein interaction networks.

### References

1. Bauer B, Mirey G, Vetter IR, *et al*. 1999. Effector recognition by the small GTP-binding proteins Ras and Ral. *J Biol Chem* **274**: 17 763–17 770.
2. Carettoni D, Gomez-Puertas P, Yim L, *et al*. 2003. Phage-display and correlated mutations identify an essential region of subdomain 1C involved in homodimerization of *Escherichia coli* FtsA. *Proteins* **50**(2): 192–206.
3. Dandekar T, Snel B, Huynen M, Bork P. 1998. Conservation of gene order: a fingerprint of proteins that physically interact. *Trends Biochem Sci* **23**: 324–328.
4. de Juan DA, Devos D, Pazos F, *et al*. 2003. Reconstruction of the *E. coli* interactome: small is still beautiful (submitted).
5. Del Sol A, Pazos F, Valencia A. 2003. Evaluation of the automatic methods for the prediction of binding sites. *J Mol Biol* (in press).
6. Enright AJ, Iliopoulos I, Kyrpides NC, Ouzounis CA. 1999. Protein interaction maps for complete genomes based on gene fusion events. *Nature* **402**: 86–90.
7. Fariselli P, Olmea O, Valencia A, Casadio R. 2001a. Prediction of contact maps with neural networks and correlated mutations. *Protein Eng* **14**(11): 835–843.
8. Fariselli P, Olmea O, Valencia A, Casadio R. 2001b. Progress in predicting inter-residue contacts of proteins with neural networks and correlated mutations. *Proteins* **45**(suppl 5): 157–162.
9. Fariselli P, Pazos F, Valencia A, Casadio R. 2002. Prediction of protein–protein interaction sites in heterocomplexes with neural networks. *Eur J Biochem* **269**(5): 1356–1361.
10. Gaasterland T, Ragan MA. 1998. Constructing multigenome views of whole microbial genomes. *Microb Comp Genomics* **3**(3): 177–192.
11. Gobel U, Sander C, Schneider R, Valencia A. 1994. Correlated mutations and residue contacts in proteins. *Proteins* **18**(4): 309–317.
12. Gomez SM, Lo SH, Rzhetsky A. 2001. Probabilistic prediction of unknown metabolic and signal-transduction networks. *Genetics* **159**(3): 1291–1298.
13. Marcotte EM, Pellegrini M, Ng HL, *et al*. 1999a. Detecting protein function and protein–protein interactions from genome sequences. *Science* **285**(5428): 751–753.
14. Marcotte EM, Pellegrini M, Thompson MJ, Yeates TO, Eisenberg D. 1999b. A combined algorithm for genome-wide prediction of protein function. *Nature* **402**(6757): 83–86.
15. Olmea O, Rost B, Valencia A. 1999. Effective use of sequence correlation and conservation in fold recognition. *J Mol Biol* **293**(5): 1221–1239.
16. Overbeek R, Fonstein M, D'Souza M, Pusch GD, Maltsev N. 1999. The use of gene clusters to infer functional coupling. *Proc Natl Acad Sci USA* **96**(6): 2896–2901.
17. Pazos F, Helmer-Citterich M, Ausiello G, Valencia A. 1997. Correlated mutations contain information about protein–protein interaction. *J Mol Biol* **271**(4): 511–523.

Copyright © 2003 John Wiley & Sons, Ltd. 

18. Pazos F, Valencia A. 2001. Similarity of phylogenetic trees as indicator of protein–protein interaction. *Protein Eng* **14**: 609–614.
19. Pazos F, Valencia A. 2002. *In silico* two-hybrid system for the selection of physically interacting protein pairs. *Proteins* **47**: 219–227.
20. Pellegrini M, Marcotte EM, Thompson MJ, Eisenberg D, Yeates TO. 1999. Assigning protein functions by comparative genome analysis: protein phylogenetic profiles. *Proc Natl Acad Sci USA* **96**: 4285–4288.
21. Rain JC, Selig L, De Reuse H, *et al.* 2001. The protein–protein interaction map of *Helicobacter pylori*. *Nature* **409**: 211–215.
22. Sprinzak E, Margalit H. 2001. Correlated sequence-signatures as markers of protein–protein interaction. *J Mol Biol* **311**(4): 681–692.
23. Valencia A, Pazos F. 2002. Computational methods for the prediction of protein interactions. *Curr Opin Struct Biol* **12**: 368–373.
24. Zuckerkandl E, Pauling L. 1965. Evolutionary divergence and convergence in proteins. In *Evolving Genes and Proteins*, Bryson V, Vogel HJ (eds). Academic Press: New York; 97–166.

Copyright © 2003 John Wiley & Sons, Ltd. 
